# The Southern Ocean mixed layer and its boundary fluxes: fine-scale observational progress and future research priorities

**DOI:** 10.1098/rsta.2022.0058

**Published:** 2023-06-26

**Authors:** Sebastiaan Swart, Marcel D. du Plessis, Sarah-Anne Nicholson, Pedro M. S. Monteiro, Lilian A. Dove, Sandy Thomalla, Andrew F. Thompson, Louise C. Biddle, Johan M. Edholm, Isabelle Giddy, Karen J. Heywood, Craig Lee, Amala Mahadevan, Geoff Shilling, Ronald Buss de Souza

**Affiliations:** ^1^ Department of Marine Sciences, University of Gothenburg, Gothenburg, Sweden; ^2^ Department of Oceanography, University of Cape Town, Rondebosch, South Africa; ^3^ Southern Ocean Carbon-Climate Observatory, CSIR, Cape Town, South Africa; ^4^ Environmental Science and Engineering, California Institute of Technology, Pasadena, CA, USA; ^5^ Centre for Ocean and Atmospheric Sciences, School of Environmental Sciences, University of East Anglia, Norwich, UK; ^6^ Applied Physics Laboratory, University of Washington, Seattle, WA, USA; ^7^ Woods Hole Oceanographic Institution, Woods Hole, MA, USA; ^8^ Earth System Numerical Modeling Division, National Institute for Space Research (INPE), Cachoeira Paulista, Brazil

**Keywords:** Southern Ocean, mixed layer, air-sea fluxes, fine-scale, autonomous platforms, submesoscale

## Abstract

Interactions between the upper ocean and air-ice-ocean fluxes in the Southern Ocean play a critical role in global climate by impacting the overturning circulation and oceanic heat and carbon uptake. Remote and challenging conditions have led to sparse observational coverage, while ongoing field programmes often fail to collect sufficient information in the right place or at the time-space scales required to constrain the variability occurring in the coupled ocean-atmosphere system. Only within the last 10 years have we been able to directly observe and assess the role of the fine-scale ocean and rapidly evolving atmospheric marine boundary layer on the upper limb of the Southern Ocean's overturning circulation. This review summarizes advances in mechanistic understanding, arising in part from observational programmes using autonomous platforms, of the fine-scale processes (1–100 km, hours-seasons) influencing the Southern Ocean mixed layer and its variability. We also review progress in observing the ocean interior connections and the coupled interactions between the ocean, atmosphere and cryosphere that moderate air-sea fluxes of heat and carbon. Most examples provided are for the ice-free Southern Ocean, while major challenges remain for observing the ice-covered ocean. We attempt to elucidate contemporary research gaps and ongoing/future efforts needed to address them.

This article is part of a discussion meeting issue 'Heat and carbon uptake in the Southern Ocean: the state of the art and future priorities'.

## 1. Introduction

The Southern Ocean buffers the impacts of climate change by accounting for 43% ± 3% of the ocean's total uptake of anthropogenic CO_2_ emissions (e.g. [1,2]) and 75% ± 22% of the heat uptake [3,4]. The large-scale anthropogenic and natural CO_2_ fluxes in the Southern Ocean are influenced mainly through the physical solubility and biological carbon pumps respectively, which are heavily impacted by the mixed layer physics and biogeochemical cycling [3,5,6]. Most of this influence occurs in the Antarctic Circumpolar Current (ACC), which has long been known to be concentrated in a number of fronts defined by enhanced meridional gradients of water column properties that extend from the sea surface to the sea floor (e.g. [7]). High-resolution hydrography (e.g. ship measurements) and satellite-derived sea surface temperature (SST), coupled with satellite altimetry and surface drifters, have made it clear that the frontal structure of the ACC is more complex than originally thought and characterized by multiple filaments and jets [8]. Research over the last decade has highlighted the important roles of mesoscale eddies, that range from regional processes that transport and mix heat and nutrients [9,–11] to global processes such as the Meridional Overturning Circulation (MOC [12]).

**Figure 1.**
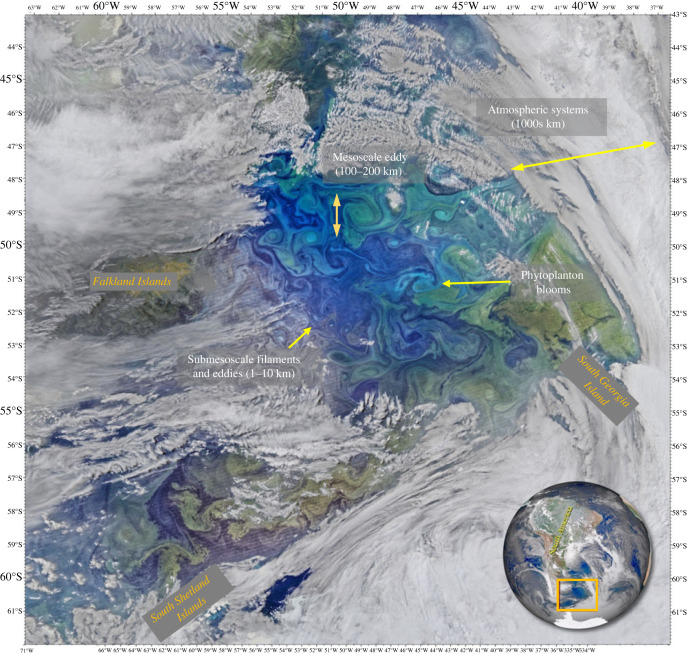
The Southern Ocean fine-scale: visible spectrum image observed by the Visible Infrared Imaging Radiometer Suite (VIIRS) aboard NASA-NOAA's Suomi NPP satellite on 16 November 2015. In this view, springtime phytoplankton blooms and aspects of the marine biome are shown swirling in various patterns in the South Atlantic Ocean between the Falkland Islands and South Georgia Island. These blooms (green to blue shades) act as tracers that highlight various ocean fine-scale dynamics, including fronts, eddies and filaments interacting between the meso- to submesoscale (space scales indicated on the image). Sea and land ice (just visible in the southern domains) perturbate the freshwater gradients in the ocean, which can act to alter ocean stratification and lateral buoyancy gradients, thereby directly impacting fine-scale ocean energetics. Overlying the ocean is a turbulent atmosphere of rapidly evolving cloud cover, winds and air masses that directly impact the coupling of the ocean to the lower marine boundary layer and associated air-sea fluxes. *Image Credit: NASA/Ocean Biology Processing Group, NASA Goddard Space Flight Center/ NASA-NOAA Suomi NPP*. (Online version in colour.)

There is growing emphasis on understanding the role of ocean dynamics occurring at the mesoscale and the smaller submesoscale on both the global ocean and climate, and its associated variability and change. For the purpose of this review, the term ‘fine-scale’ is used to encompass processes associated with the ocean circulation occurring at spatial scales from the submesoscale (0.1–10 km and Rossby numbers of O(1)) to the mesoscale (10–200 km) with temporal scales of days to months (intra-seasonal to seasonal) depending on their size, latitude and how they are impacted by their surrounding environment. Typically, the ocean fine-scale is characterized by circulation features resembling eddies, fronts, meanders and filaments that are associated with enhanced vertical transport connecting the ocean's mixed layer to the interior [13]. They help to shape ocean circulation and the marine biome up to the climate scale [14,15]. Due to the content of this review, we feel the need to incorporate into the fine-scale the influence of atmospheric circulation given how it modifies the characteristics of the atmospheric marine boundary layer. In particular, we require consideration of the role of synoptic storms that rapidly modify surface winds, air masses and air-sea fluxes that promote coupling and feedbacks [16] between the ocean fine-scale and the atmosphere. Figure 1 encapsulates these various interacting processes and their length scales using a single satellite image from the Southern Ocean.

Such processes are not captured in global climate models, and it is yet to be determined how substantially fine-scale processes impact climate projections [17]. The Southern Ocean is particularly important in this respect, not least due to its disproportionate role and sensitivity to climate variability and change [18,19], but also because it is associated with high mesoscale activity, has large domains covered by seasonal sea ice (18 million km^2^) and is impacted by immense atmospheric storms (approx. 1000 km; [20]). These processes impact the surface ocean boundary layer and thereby the exchange of heat and carbon between the ocean interior and the atmosphere. A major challenge is to extract the potential role of fine-scale processes on the longer-term climate perturbations that are projected in the coming century. Addressing this need requires innovative experimental designs, fine-scale observations, and satellite missions together with high-resolution coupled models [21,–23] that can adequately interrogate the physical-biological and ocean-atmosphere coupling of processes in this unique and globally important part of the ocean.

This review provides an overview of the progress in understanding fine-scale ocean variability gleaned from observations collected by autonomous profiling gliders and surface ocean platforms, profiling floats, ships and from the network of Elephant seal-tag observations in the Southern Ocean. These platforms have made a step-change in our view of the Southern Ocean by resolving the variability of processes with spatial scales of O(1–100 km) and temporal scales ranging from hours to seasons. Fieldwork focusing on Southern Ocean fine-scales has primarily adopted a process study approach, and here we aim to collate the collective learning of these efforts to:

1. Contrast processes that impact mixed layer variability in space (latitudinally, over changing topography, regionally) and over a range of environmental conditions (sea ice, storms, seasonal cycle of surface buoyancy).
2. Elucidate how the Southern Ocean mixed layer couples with the ocean interior, atmosphere and cryosphere to modulate fluxes of heat, freshwater and carbon at the fine-scale.
3. Identify challenges in our current observational capability to observe fine-scale processes and highlight perceived research gaps and potential solutions to address them in the future.

**Figure 2.**
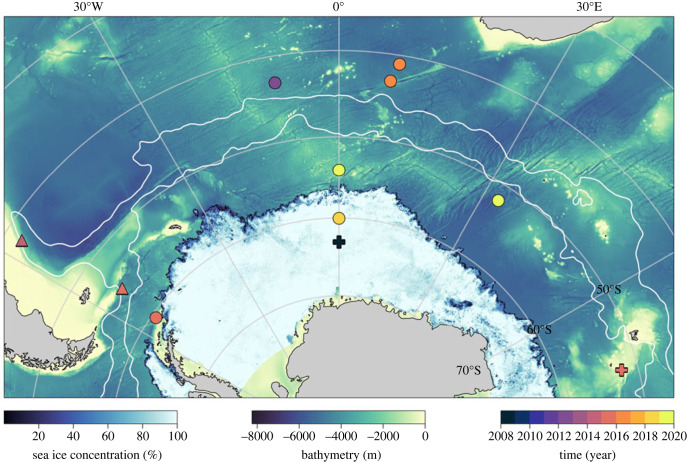
Geographical distribution of observational campaigns summarized in this review. Solid contours represent, from north to south, the Subantarctic Front and the Polar Front of the Antarctic Circumpolar Current. The sea ice extent (1 August 2020) is indicated providing a view of the sea ice distribution during austral winter. The various observational campaigns are coloured by year of data collection and overlaid onto the bathymetry. Dots represent glider surveys, triangles are ship-based observations and plus-signs represent Elephant seal-tag datasets that are presented in this review. (Online version in colour.)

A concentration of instruments suited to observe the ocean fine-scale and associated air-sea fluxes have been deployed in the Subantarctic to Subpolar and sea ice regions of the Southern Ocean, south of Africa. This has an obvious bias of presenting the findings associated with the South Atlantic. Where possible, this is balanced by studies conducted in Drake Passage, Brazil-Malvinas Confluence, near the Kerguelen Plateau and in the Indian Sector open ocean (figure 2). Nonetheless, the processes described here, covering regions of both low and high eddy kinetic energy (EKE), are expected to be ubiquitous, and their impacts relevant to most of the Southern Ocean and even globally.

## 2. The role of fine-scale physics in driving mixed layer variability and associated biological response

Multiple underwater ocean glider deployments were initiated in the Subantarctic region (Subantarctic Zone, SAZ, figure 2) of the Southern Ocean in 2012 to provide an *in situ* assessment of the drivers of the satellite-based intraseasonal chlorophyll variability reported in Thomalla *et al*. [24]. These deployments observed, for the first time, the variability in mixed layer depth (MLD) and chlorophyll response, at sub-daily resolution, during the spring to late summer re-stratification period that supports the seasonal phytoplankton bloom [25].

Although glider deployments open up new avenues for sampling time and space scales that were previously inaccessible, the slow propulsion of gliders through a varying field of the ocean in space and that evolves with time adds complexity to the interpretation of their datasets. For example, Rudnick *et al*. [26] suggested that temporal and spatial variability can be complex to distinguish in ocean glider data because they transect through various water masses by crossing fronts and eddies, while at the same time, observing the temporally adjusting environment brought on by various processes, such as synoptic storm-driven mixing. Little *et al*. [27] used one of the initial Subantarctic glider deployments at the Prime Meridian to discern how much of the variability measured by a glider was the result of temporal variations versus spatial patchiness. Their study compared the high-resolution glider dataset (3 h, 2 km horizontal resolution) with remotely sensed satellite data (2–4 km) to reveal that the scales of chlorophyll adjustments are primarily driven by fine-scale ocean physics, but with some important seasonal nuances. The springtime chlorophyll variability primarily occurs at timescales less than 10 days and is correlated to fine-scale variability in the physical variables of wind, MLD and SST. A likely explanation is that fine-scale processes, such as submesoscale mixed layer processes, manifest in spring during periods of weakening wind stress and positive solar heat fluxes that allow the upper ocean to restratify [28,29]. These heterogeneous restratifying processes in turn improve the light environment that can drive sporadic and patchy blooms in nutrient replete environments [25]. During summer, the observed mixed layer chlorophyll variability shifts towards both monthly and 10-day modes matched by the physical variables, which is compatible with the proposed driver being synoptic storm events that pass through the region, changing the MLD and phytoplankton growth rates [29,30].

**Figure 3.**
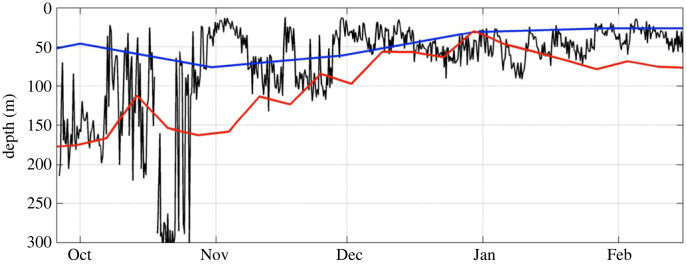
Vertical extent of the mixed layer at fine-scale: prior to high-resolution upper ocean observations in space and time, the community was mostly reliant on gridded monthly or weekly products of temperature and salinity profiles in order to derive metrics like MLD. The 2012–2013 austral spring to summer MLD estimates from two gridded/reanalysis products are shown: monthly EN3 in blue and weekly CFSR in red. These coarse MLD estimates are made at the same location as a Seaglider deployed during September 2012 to February 2013 (SOSCEx; [31]). The glider-derived MLD estimates are shown with the black line where the average temporal resolution of the glider data is 2.5 h. The glider time series of MLD resolves significantly higher-frequency variability in the MLD as well as observing a substantially larger range of MLD values as compared to EN3 and CFSR products. (Online version in colour.)

The Subantarctic glider deployments of 2012 provided the first datasets for the open-ocean Southern Ocean that were able to address the meso- to submesoscale and seasonal to intra-seasonal timescales necessary to bridge important gaps in our understanding of the ocean carbon-climate system. In particular, the five month deployments of two gliders in the Subantarctic revealed upper ocean lateral buoyancy gradients that varied regularly over short time and space scales, confirming the importance of fine-scale features (meso- to submesoscale) in dictating the physical environment of the upper 300 m of the ocean [25]. Springtime periods of larger horizontal temperature, salinity, and density gradients, and mixed layers extending to approximately 300 m depth, underwent intermittent shoaling events (MLD ∼ 20–50 m) lasting several days before deepening again to 100s of metres (MLD mean 80 ± 52 m, figure 3). In summer, the MLD remained highly variable (20–90 m) at short time scales (approx. 7 days) but with maximum depths constrained by enhanced stratification (from solar heat flux) that limits the wind-driven deepening of the MLD to approx. 40 m. The observed rapid shoaling of the MLD in summer (greater than 40 m day^−1^) was facilitated by the presence of horizontal density gradients (baroclinic shear) associated with meso- to submesoscale eddies and fronts that act to rearrange buoyancy [32] and amplify surface restratification on the order of days. A net effect is to alter the light availability and nutrients in the upper ocean at appropriate time scales for phytoplankton growth, thereby sustaining the bloom for an extended period through to late summer [33].

Autonomous platforms have also provided insight into the spatial decorrelation scales of fine-scale MLD variability. For instance, an assessment of data from two gliders sampling concurrently during the summer (November to mid-February), separated by up to 213 km, show simultaneous MLD deepening and shoaling, supporting the view that intra-seasonal MLD variability during summer is primarily driven by synoptic scale winds occurring at meso- to large scales (100–1000 km) (see electronic supplementary material in [25]). However, differences in the MLD (100–250 m) observed between the two gliders were significantly greater in austral spring (September–October). These differences likely reflect increased variability of the MLD driven by submesoscale mixing and restratification processes, which are prominent in the presence of weak stratification and strong lateral buoyancy gradients enhanced by mesoscale stirring [32,34].

A follow-on study by du Plessis *et al*. [29] proposed that rapid springtime restratification events are due to submesoscale mixed layer eddies (MLE; [28]). du Plessis and co-authors showed that during periods of low winds, an instantaneous equivalent heat flux (an estimate of a heat flux magnitude that would be required to restratify the surface ocean at the same rate as the submesoscale eddies) can be comparable to the local air-sea heat fluxes of approximately 500 W m^−2^. However, the newly restratified mixed layer may erode over timescales of days during periods of increased wind stress, assisted by an Ekman buoyancy flux (EBF), where winds directed along the flow of a front oppose MLE restratification through an Ekman transport of water from the dense side of the front over the lighter side. Equivalent heat fluxes for the wind-driven EBF regularly exceed those of MLEs in the Subantarctic, where westerly winds blowing over the ocean provide the optimal wind-front alignment for EBF mixing to occur.

Concentrated efforts to deploy gliders in the same Subantarctic region (43° S, 8.5° E, figure 2) for four consecutive years during the winter-spring-summer period allowed for an inter-annual investigation into the role of submesoscale dynamics on the seasonal restratification of the mixed layer [35]. In contrast to the du Plessis *et al*. [29] findings, the restratification of the mixed layer occurred up to two months *after* the onset of seasonal surface heat flux warming, with destabilizing EBF equivalent heat fluxes estimated to be large enough to reverse the net ocean restratification flux and cause the observed delay in restratification. Simple parameterization of the submesoscale equivalent heat flux in a one-dimensional mixed layer model indicated that the EBF reduces the magnitude of summertime stratification by a factor of 2 when compared to the one-dimensional model run with surface heat flux forcing alone [35].

Ultimately, deep Southern Ocean mixed layers together with down-front winds (i.e. mean ACC currents that are aligned with the prevailing westerly winds) provide a continuous interplay (tango) between MLE and EBF processes that modulate the ocean stratification at scales of a few kms and the synoptic storm period (approximately weekly). This is particularly the case during the autumn–winter-spring seasons when stratification is weak, MLDs are deep (i.e. larger available potential energy) and surface winds are stronger. Further glider missions and one-dimensional model simulations, with submesoscale parameterizations included, were conducted in Drake Passage [36], a region of significantly enhanced eddy kinetic energy (EKE). Their findings reveal larger lateral buoyancy gradients than observed in the SAZ and highlighted a more prominent role of topography on MLE restratification that causes enhanced MLD variability. A comparison of these two regions (Subantarctic and Drake Passage) suggests that submesoscale processes are at least as important as surface wind and buoyancy fluxes in governing mixed layer variability in the Southern Ocean.

The interplay between intra-seasonal mixed layer deepening and shoaling events alters upper ocean euphotic conditions and nutrient supply with strong implications for biological productivity. This subseasonal MLD variability was proposed to enable sustained phytoplankton blooms over the entire summer period of approximately three months of glider observations in the Subantarctic [25]. Eddy resolving physical-biogeochemical model simulations have since shown the potential for iron and other nutrients to be entrained into the upper ocean and laterally stirred by fine-scale ocean and storm-driven processes [37,38]. In particular, the interactions between storms and the underlying ocean fronts in these simulations results in enhanced summertime dissolved iron supply via diffusive [30] and advective processes beyond the ‘once off' winter supply by deep mixing [39] and can stimulate primary production by 20% [37]. Such intra-seasonal processes have a dominant and widespread imprint on surface phytoplankton production across the Southern Ocean as revealed by satellite ocean colour observations [24,40,41].

Submesoscale processes have been predicted to contribute up to 50% of the springtime carbon export in the Southern Ocean through episodic injections along sloping isopycnals, a process termed the eddy-subduction pump (observations from the North Atlantic shown in [42]). Further, Stuckel & Ducklow [43] estimated that vertical mixing and subduction of organic matter, including episodic subduction, could represent 23% of the total biological carbon pump in the Southern Ocean. The eddy-subduction pump accounts for mixed-layer instability in the surface ocean associated with fronts, driving particle-rich surface waters to depth. The eddy-subduction pump is expected to contribute most to carbon fluxes when mixed layers are deep and particulate organic carbon at the surface is high; these conditions are met at the same time under spring bloom conditions [44].

Measuring particulate carbon fluxes with autonomous vehicles remains challenging as a result of separating the contribution of spatial and temporal variability in a quasi-Lagrangian platform as well as the limitations in measuring vertical velocities. Llort *et al*. [45] uses a limited parameterization that locates anomalies in density compensation and oxygen below the base of the mixed layer to estimate the values of the eddy-subduction pump from BGC-Argo floats across the Southern Ocean, finding that such localized subduction events actually contribute only 1% of carbon export. However, they found that these events may contribute more to total carbon export in summer months (up to 40%).

## 3. The effects of topography on submesoscale physics and connections to the ocean interior

The topography of the Southern Ocean modulates key dynamical features of the ocean's flow, including EKE [46] and vorticity [47]. Recent high-resolution observational efforts indicate that topography, largely through the generation of standing meanders and modulation of mesoscale kinetic energy in the ACC, additionally has a significant impact on the regional inhomogeneity of submesoscale energetics and their effects on MLD variability. Before sampling by gliders and other high-resolution techniques became commonplace, the impact of topography on processes at the submesoscale was predicted by numerical models. Rosso *et al*. [48] demonstrated spatial inhomogeneity in submesoscale activity correlated with topography in a submesoscale-resolving (1/80° resolution) ocean model with realistic bathymetry of the Kerguelen Plateau; vertical velocities were enhanced in the lee of topography, often exceeding many tens of metres per day. Meanwhile, Balwada *et al*. [49] used an ACC-like channel model with a meridional ridge and Backman & Klocker assess several simulations at the Kerguelen Plateau between 1/4° and 1/120° to study the impact of submesoscale-resolving simulations on tracer fluxes. The submesoscale-resolving (approx. 1 km) resolution simulations have significantly deeper-reaching and stronger fronts, particularly downstream of the ridges, that lead to large vertical fluxes. These higher resolution models can dramatically increase vertical tracer transport by several orders of magnitude compared to solely mesoscale-resolving simulations, a process that is sensitive to varying wind stress (see [50]).

**Figure 4.**
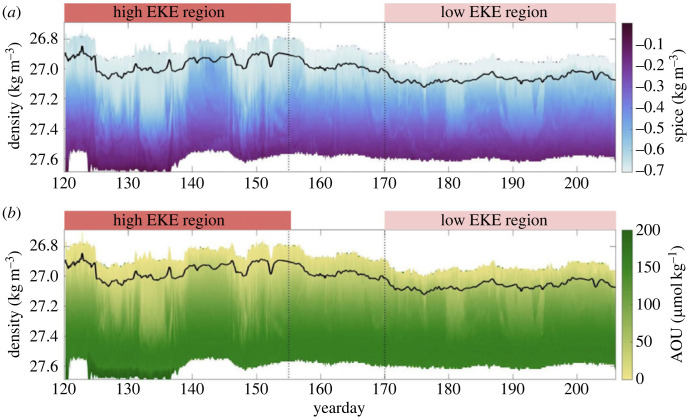
Variability in tracer distribution at the submesoscale in high and low EKE regions: time series of (*a*) spice (kg m^−3^) and (*b*) AOU (μmol kg^−1^) from a Seaglider mapped onto potential density surfaces at the Southwest Indian Ridge (51° S, 33° E). Black line denotes the MLD. Dotted line at yearday 155 denotes an end to the high-eddy kinetic energy (EKE) region and the dotted line at yearday 170 denotes the start of the low-EKE region. Figure adapted from Dove *et al*. [51]. (Online version in colour.)

Observations have also indicated that topographic features, even when the sill depth is well below the ocean surface, can mark abrupt transitions in mixed layer properties and their variability. Glider observations collected from the strong mesoscale eddy field of the Drake Passage show that MLD, variability and lateral buoyancy gradients transition across the Shackleton Fracture Zone [36]. MLDs were approximately twice as deep, and lateral buoyancy gradients were substantially higher downstream of the fracture zone. This change is largely induced by the large-scale displacement of the Polar Front as it traverses the fracture zone; upstream the fronts are strong and largely zonal, such that EKE is relatively weak. The front meanders downstream, generating stronger mesoscale eddies that effectively stir the background density gradient. Dove *et al*. [51] focus on the downstream transition between high and low EKE regions using measurements from floats and gliders advected through an ACC standing meander formed by the interaction of the Polar Front with the Southwest Indian Ridge. In the high EKE standing meander, submesoscale gradients are stronger compared with further downstream. These enhanced gradients are correlated with greater tracer variance of apparent oxygen utilization (AOU) and spice (figure 4). This enhanced variance extends below the mixed layer and into density surfaces associated with Upper Circumpolar Deep Water. This suggests the active transport and ventilation of waters between the surface and ocean interior (peak tracer variance at 350 m depth) beyond a one-dimnensional view of vertical sinking of particulate matter. The standing meanders are also characterized by increased tracer variance both in the surface mixed layer and along isopycnals at intermediate depths than nearby semi-Lagrangian floats, suggesting that the repeat profiling of Argo floats maximizes the temporal and spatial coverage of the dataset, but comes at the cost of aliasing small-scale dynamics. However, the work of Llort *et al*. [45] suggests that float profiles can detect the signature of submesoscale vertical velocities as co-located low spice, low AOU, and high chlorophyll anomalies below the base of the mixed layer, linked to submesoscale subduction events. Strikingly, these anomalies are almost exclusively found in high EKE regions downstream of three Southern Ocean bathymetric features: the Kerguelen Plateau, southeast Indian Ridge and the Eastern Pacific Rise.

While this review focuses on the surface signature of fine-scale variability, with a view towards its potential impact on air-sea exchange, fine-scale variability is not limited to the surface boundary layer. Data from instrumented elephant seals downstream of the Kerguelen Plateau show deep-reaching submesoscale fronts (greater than 300 m) that drive a localized, anomalous upward heat transport in this energetic region with a magnitude comparable to instantaneous air-sea fluxes [52]. These findings indicate the key role that underlying bathymetry plays in the generation of fine-scale variability over limited spatial scales and their importance to correctly understand the drivers of upper-ocean stratification.

A key message arising from these studies is that fine-scale variability occurs due to broad and potentially complex interactions between submesoscale and mesoscale circulation features, which shape tracer distributions across the Southern Ocean. Strain fields associated with coherent mesoscale eddies have an important role in generating and modifying deep-reaching submesoscale fronts [53,54]. Indeed, the spatial heterogeneity of submesoscale processes across the Southern Ocean are, to first order, predicted by mesoscale EKE. In particular, the EKE and strain rate can be used as a proxy for submesoscale vertical velocity [48], and observationally using finite-size Lyapunov exponents (FSLEs). Siegelman *et al*. [52] describe an empirical relationship between FSLE values computed from satellite-derived geostrophic velocities and observed lateral gradients of buoyancy at 300 m. Peaks of FSLE and buoyancy gradients are often co-located, showing that an eddy strain field, observed at the sea surface, can drive strong buoyancy gradients. Dove *et al*. [55] extended this work to illustrate that FSLE values are enhanced downstream of the major flow-topography interactions across the ACC. While submesoscale vertical velocities in the surface ocean may lead to the transport of anomalies to the base of the mixed layer, lateral stirring by the mesoscale contributes to the setting of observed tracer distributions.

An emerging research area seeks to explore how the ACCs regional variability, at the scale of standing meanders, impacts temporal fluctuations in air-sea fluxes. Previous work on air-sea carbon fluxes has emphasized the role of the larger-scale structure of the westerly wind field, and in particular its meridional component, on interannual CO_2_ flux changes [3]. However, variations in the shape of individual standing meanders have been shown to strongly impact momentum and energy budgets of the ACC [56,57] as well as the Southern Ocean overturning [58]. This meander ‘flexing' may also modulate surface fluxes, but to date observations have been sparse in these regions and until this is resolved, high-resolution coupled models are likely to provide crucial insight.

## 4. The influence of sea ice on mixed layer and submesoscale processes

The Antarctic seasonally sea-ice-covered ocean remains one of the least observed systems on the planet, with limited access rendering it arguably the largest ‘blind spot' of current global ocean-climate research. The growth, melt and movement of sea-ice, in combination with its complex interaction with the ocean and atmosphere and influence on air-sea fluxes, make this area of research particularly complex to observe and understand. Antarctic sea ice is comparatively thin compared to Arctic sea ice and is thereby generally unable to support Arctic-designed instrumentation, such as drifting Ice Tethered Profilers capable of collecting relatively high-resolution hydrographic profiles within sea ice over many months at a time [59,60]. Most of our understanding on the feedbacks between submesoscale processes and the presence of sea ice rely on Arctic-based modelling studies and observations (e.g. [61]). However, modelling studies that specifically consider Southern Ocean/ACC conditions with sea ice have recently emerged [62] and indicate the importance of the mesoscale eddy field interaction with sea ice flows to induce vertical velocities in the upper ocean [63]. It has been simulated that the growth or melt of sea ice introduces mixed layer buoyancy gradients that spur the development of submesoscale eddies and associated vertical heat transport towards the surface [64–66].

The seasonal growth and melt of Antarctic sea ice (representing a surface area of approx. 18 million km^2^) results in vast amounts of freshwater flux (melt or growth) to the mixed layer and drastically varies the area of ocean exposed to the atmosphere, modulating air-sea exchanges of heat, gases and momentum [67]. Compared with the Arctic, Southern Ocean sea ice extends to considerably lower latitudes (55° S) and is exposed to stronger surface forcing, such as storm passages within the Southern Hemisphere westerly wind belt.

The first comprehensive observational effort to observe the effects of sea ice melt at the fine-scale in the Southern Ocean was completed in 2018 and characterized the evolving mixed layer physics and submesoscale dynamics over the austral summer. This study by Swart *et al.* [68] used several glider deployments together with an autonomous surface vehicle (Sailbuoy) during spring sea ice melt and extended for over three months into austral summer. Prior to this, evidence for the magnitude and variability of lateral density gradients within the mixed layer and associated instabilities occurring in the Antarctic Marginal Ice Zone (MIZ) were assumed from high-resolution Sentinel-SAR imagery, where signatures of 5–10 km-scale ocean eddies and filaments were apparent in the disintegrating sea ice [68].

During austral summer, the rapid and widespread melt of Antarctic sea ice introduces large-scale (O(100 km)) freshwater to the surface layer. This freshwater is stirred by mesoscale motions that can generate sharp salinity fronts through frontogenesis [68]. These fronts store potential energy that fuel the growth of submesoscale motions and baroclinic instabilities observed by gliders and the Sailbuoy occurring at a range of horizontal scales from 0.1 to 100 km [68,69]. Following sea ice melt, mixed layer density fronts, together with shallow mixed layers, are highly responsive to mechanically driven mixed layer EBF (greater than 200 W m^−2^). These strongly coupled atmosphere-ocean interactions maintain submesoscale variability weeks after the melt of sea ice [68]. However, the shallow MLD and high stratification at the base of the mixed layer limit the potential energy available for the growth of deep reaching baroclinic MLEs [69]. This region is therefore prone to reduced submesoscale-driven ocean interior/mixed layer exchange of water properties compared to those observed in more energetic and weakly stratified regions of the Southern Ocean further north [35,36,51].

The summer evolution of submesoscale fronts following sea ice melt was investigated with density variance spectra, used as a proxy for potential energy spectra. Density variance spectra scale with inertial slopes of *k*^−2^ (where *k* is the horizontal wavenumber), predicted when surface quasi-geostrophic theory is adjusted for ageostrophic submesoscale motions [13]. The observations obtained in early summer sea ice melt period indicate slopes of *k*^−2.4^ that steepen to *k*^−3^ in late summer resembling an ocean more dominated by mesoscale motions. This trend towards steeper slopes is likely a result of the dispersion of meltwater-derived fronts by submesoscale wind-front interactions and the cessation of meltwater-derived fronts following the complete seasonal thaw of sea ice [69].

Although recent observations from spring-summer deployments of autonomous platforms in the Antarctic seasonal ice zone are beginning to shed light on the complex dynamics that govern the region during largely ice-free conditions, the autumn and winter months remain under-sampled and less understood. The first observations to provide insight into the wintertime under-ice activity of submesoscale motions came from elephant seal-tagged hydrographic datasets [70]. Data were collected between January to November with modal temporal frequency of 6 h and spatial resolution of 9 km. These datasets are relatively low resolution compared with those collected from gliders, for example, and represent a lower-order magnitude of lateral buoyancy gradients occurring at the submesoscale. Despite a reduction in air-sea interactions due to the presence of sea ice, it was found that the potential for MLEs to be present was highest during the austral winter (August/September) when the MLD deepens and lateral buoyancy gradients increased, possibly due to brine rejection during sea ice formation. This highlights the potential to restratify the ocean under sea ice and in winter, thereby impacting mixed layer properties and the magnitude of air-ice-ocean fluxes.

## 5. Coupling between the fine-scale ocean and air-ocean-ice fluxes of heat, carbon and fresh water

The distinctive surface ocean conditions of the Southern Ocean, including strong and variable winds, sea state, relatively sharp temperature gradients and large seasonal changes of sea ice cover, allow for enhanced air-sea exchanges in a region critical to global climate and highly responsive to our changing climate [71,72]. The Southern Ocean's characteristically weak stratification, deep mixed layers [73] and steeply tilted isopycnals mean that relatively small energy inputs can mix the ocean vertically, thereby increasing the potential to transport properties between the deeper ocean interior, surface ocean and to the lower atmosphere via air-sea exchange. The air-sea exchanges can feedback to the surface ocean by altering buoyancy and stratification to enhance or arrest mixing processes and are thus essential to thermocline ventilation [74].

**Figure 5.**
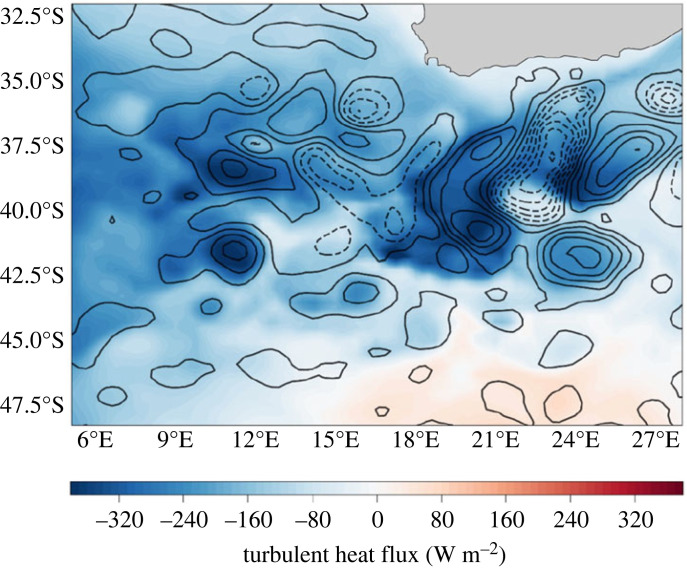
The ocean's mesoscale imprint on the air-sea heat flux: a low-resolution (25 km) ‘snapshot' of reanalysis turbulent heat flux (in colour—ERA5) mirroring the mesoscale ocean circulation underneath for the Subantarctic—Subtropical domain south of Africa. Satellite sea surface height contours (0.2 dyn m contour spacing with solid lines for positive and dashed for negative SSH anomalies) define this ocean circulation and can be seen as closely correlated to the air-sea heat flux. Similar views are provided by Chelton & Xie [81]. (Online version in colour.)

Direct observations of the fine-scale ocean-atmospheric coupling of energy and property pathways remain a significant knowledge gap, particularly in the Southern Ocean. Coupled models suggest the remarkable role of the fine-scale ocean, with the sharpening and increasing intensity of SST fronts extending down to the km-scale modulating the surface heat fluxes and wind stress divergence [75,–77] and thereby energizing atmospheric storms [78]. Ongoing observational evidence, often in subtropical western boundary currents, has focused on the role of the mesoscale anomalies in driving strong coupling with the atmosphere [79], at times being responsible for 50% of latent heat fluxes [80]. Such evidence of the ocean's mesoscale circulation imprint on the air-sea turbulent heat fluxes [81] can be seen qualitatively in ERA5 reanalysis data [82] in the northern domains of the Southern Ocean, particularly at boundaries to western boundary currents, such as the Agulhas Current (figure 5). Mesoscale ocean eddies have also been shown to have direct implications for air-sea gas fluxes. Pezzi *et al*. [83] described that a warm-core eddy ejected from the Brazil Current towards the adjacent Subantarctic waters is capable of changing the CO_2_ fluxes to outgas, in contrast to neighbouring waters that are sinks of CO_2_. Agulhas mesoscale rings translating across the Atlantic Ocean can, on the other hand, enhance ocean carbon uptake by 2.5 tons CO_2_ in an eddy lifetime [84].

**Figure 6.**
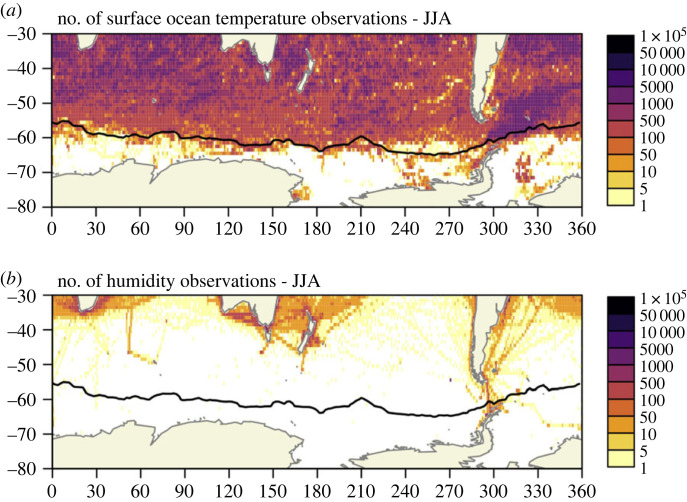
Data coverage over the Southern Ocean: 10-year (2008–2017) *in situ* observation count for critical air-sea flux ocean and atmospheric parameters during austral winter (June–August). As an example, (*a*) near surface ocean temperature (0–5 m depth) shows comparatively broad distribution in spatial coverage compared with (*b*) lower atmosphere humidity observations, which are extremely sparse and limited mostly to ship tracks. Maps generated from International Comprehensive Ocean-Atmosphere DataSet (ICOADS), as described by Freeman *et al*. [85]. Black contour denotes the 10-year mean of the 15% sea ice concentration from NCEP/DOE AMIP-II Reanalysis, representing the sea-ice edge in winter. Figure adapted from Swart *et al*. [86]. (Online version in colour.)

The observations required for estimating air-sea-ice fluxes at the ocean/ice surface are severely lacking in the Southern Ocean, with no autumn–winter observations recorded over large swaths of its domain (figure 6). This leads to large errors in commonly used reanalysis products for winds and heat [86,87] and in representations in climate models due to a lack of knowledge of the strongly coupled ocean-atmosphere dynamics [88,–90]. Poor observational coverage is largely driven by the difficulty in acquiring the measurements needed to estimate fluxes (e.g. pCO_2_, air temperature, wind speed, humidity) from instruments located at the sea surface or within the marine atmospheric boundary layer. While autonomous instruments in the ocean, namely profiling floats, provide relatively good coverage in space and time north of the winter sea ice edge (figure 6*a*), measurements needed for flux estimates have historically been reliant on ships [91] or surface moorings ([92,93]—in this issue) (figure 6*b*). More recently, the use of autonomous surface vehicles, such as Wave Gliders, Sailbuoys, Saildrones and Ice Mass Balance Buoys in sea ice, are starting to fill gaps (as detailed below) but still remain far off the required coverage to address our lack of understanding of air-sea-ice fluxes and their variability. An additional complexity is that air-sea interactions are influenced by the properties of the mixed layer and the processes which alter them, such as mixed layer deepening and entrainment. A holistic understanding of the ocean-atmosphere coupled system and its associated air-sea exchanges requires simultaneous fine-scale, high-frequency multidisciplinary observations of the upper ocean and air-sea interface, a feat only recently being realized in the Southern Ocean due, in large part, to the growing capability of autonomous platforms and sensors.

Even more critically sparse are direct measurements of air-sea fluxes from methods such as those obtained by Eddy Covariance (EC) methods. Rare high-frequency micrometeorological measurements required for EC estimates have been obtained on Brazilian research vessels (figure 2) over several years but remain otherwise elusive over the rest of the Southern Ocean. Results of Santini *et al*. [94] show that EC estimates of sensible heat fluxes compare well with those derived using bulk-formulae (difference less than 20 W m^−2^). However, bulk-derived latent heat fluxes experience regular negative differences of between 75 and 100 W m^−2^ when compared with the EC method. These were coincident with either high-frequency atmospheric perturbations, such as strong wind bursts at the air-sea interface and cold, dry air outbursts (e.g. [92]) or due to sharp SST gradients associated with the fine-scale ocean (eddies, fronts, [95]) demonstrating an inability for bulk-derived heat fluxes to take into account the important contribution of fine-scale/short-term (minutes to hours) atmosphere and ocean adjustments that change the local air-sea fluxes and marine atmospheric boundary layer stability.

Strong storms prevalent over the Southern Ocean inflict intense surface wind-stress that energizes the mixed layer, driving mixing and advection that promotes surface to subsurface ocean exchanges of heat, momentum and chemical properties. Twinned autonomous platforms, sampling the ocean and lower atmosphere in unison (profiling glider with autonomous surface vehicle, figure 2), were designed to simultaneously observe the response of the coupled air-sea and mixed layer interfaces to storms. In the Subantarctic region of the Atlantic Ocean, Monteiro *et al*. [96] used pCO_2_ measurements from Wave Gliders to explain the dominance of storm-driven intra-seasonal variability on CO_2_ air-sea fluxes, highlighting the need for higher frequency (less than 3 days) observations to avoid aliased-driven biases to the mean estimates of the Southern Ocean carbon sink. Further poleward, in the subpolar Southern Ocean (south of the Polar Front, figure 2), Nicholson *et al*. [97] showed large-scale CO_2_-rich Upper Circumpolar Deep Water (UCDW) is upwelled to the near surface ocean when strong storms drive deep mixing (dissipation measurements from glider with microstructure capability) beyond the mixed layer extent and into the subsurface reservoir of dissolved inorganic carbon-rich UCDW. This results in substantial CO_2_ outgassing events (2–4 mol m^−2^ yr^−1^). Further, they show that wind-driven advection from regular passing storms drives substantial variability in CO_2_. These results highlight the importance of intra-seasonal processes (e.g. passing storms) for understanding the present and future Southern Ocean CO_2_ sink and build on the findings that the southern domains of the Southern Ocean act as a source for CO_2_, particularly in winter [98].

Such passing storms can also link with subtropical atmospheric systems to transport significant atmospheric freshwater fluxes to the Southern Ocean. Edholm *et al*. [99] used fine-scale *in situ* data from the same Wave Glider deployments in the subpolar Southern Ocean to evaluate the impact of atmospheric rivers (ARs, [100]) on surface ocean buoyancy variability. They found that precipitation from ARs can account for 10% of the total buoyancy gained during summer, while increasing the precipitation output from passing storms by twofold. While surface warming dominates buoyancy fluxes throughout the full time series (79% of total gained during summer), the strong AR-fuelled storms produced the largest instantaneous buoyancy fluxes at daily timescales. Their study, coupled with the findings in du Plessis [101], argues that the large freshwater fluxes are vertically distributed into the mixed layer, rather than horizontally advected, thus lowering the salinity in the mixed layer over summer. Given these observations showing how storms and ARs add to surface buoyancy in the Southern Ocean, the authors argue that ARs contribute to the seasonal variability of surface buoyancy in the Southern Ocean, which are proposed to impact the sea ice conditions and polynya formation in the polar gyres [102]. Studies such as these highlight the potential role of subtropical atmospheric circulations and air masses impacting air-sea fluxes at high latitudes, adjusting energy cycles and upper ocean processes at both the regional and local scale.

## 6. Future research and observational priorities to reach a more holistic view of the fine-scale in the Southern Ocean

This review demonstrates the value of *in situ* observations to understand the role of fine-scale processes in setting the distribution and exchange of properties, such as heat and carbon, between the Southern Ocean's interior, mixed layer and across the air-sea interface. In particular, we attempt to highlight the need to observe, at high-resolution, the surface mixed layer and several interacting boundaries (ocean interior, atmosphere and cryosphere) in order to track and estimate the rate and magnitude at which properties are exchanged across these boundaries of the Earth system. Right now, observations are likely too sparse to draw definitive conclusions from the Southern Ocean, although other field programmes, such as the NASA S-MODE effort off the coast of California and the ONR CALYPSO project in the Mediterranean Sea, are beginning to access this dynamical regime. Nonetheless, recent Southern Ocean field programmes using heterogeneous arrays of autonomous platforms, e.g. combinations of gliders, floats, Wave Gliders, Sailbuoys, Saildrones and moorings (e.g. ROAM-MIZ—[68,103], SOSCEx—[97], SO-CHIC—[104]; SOLACE – [51]) show that spatially varying upper ocean properties and air-sea flux data can be acquired in tandem and with the required spatial and temporal resolution to validate and more deeply explore processes that have been studied using high-resolution coupled simulations. Although several of the research and observational challenges are embedded in this review, there are several aspects to highlight that should be prioritized to progress this field of research in the future:

— Dedicated ship surveys targeting the fine-scale, such as demonstrated in Adams *et al*. [53], have been very limited in the Southern Ocean. Ships provide major opportunities to complete fine-scale multidisciplinary studies by collecting measurements of variables and systems that are currently out of reach of autonomous platforms. In particular, ships are well adapted to make (1) detailed atmospheric and air-sea flux measurements using EC towers, lidar and radio-sounding using balloon deployments, (2) seawater sampling for biogeochemistry, such as carbonates, nutrients and isotopes, (3) observing the biology to understand plankton diversity and genomics, particulate export assessments throughout the upper water column (e.g. McLane pumps), and (4) for more detailed ocean physics measurements using undulators, vertical microstructure profilers and low-frequency ADCPs. Ships and other faster moving platforms (e.g. powered AUVs and USVs) are also superior in capturing the ‘synoptic' view of rapidly evolving processes and features (e.g. submesoscale filament or atmospheric front) compared to slower-moving gliders.
— The need for combined ocean and lower marine boundary layer observational experiments (e.g. twinned glider and surface vehicles) observing the ocean interior, mixed layer, air-sea interface and atmosphere, simultaneously. These should be targeted in seasons (autumn to winter) with previously little data/knowledge and regions subject to high variance in tracer transport between the ocean interior, air-sea interface or both (e.g. standing meanders or at the boundaries between the ACC and subtropical ocean).
— Machine learning/artificial intelligence techniques are becoming prominent and opening prospects for adaptive and ‘self-aware' [105] autonomous multi-platform sampling, which is particularly important given the lognormal spatial distribution of features like ocean fronts [106]. Realistic and operational high-resolution model simulations could better inform field campaigns (e.g. [107]) to target specific features and dynamics to capture the spatial variability of fine-scale ocean dynamics. Also, Observing System Simulation Experiments (OSSEs) are valuable in determining the amount of variance captured given the regional or temporal placement of certain observing platforms [108] to inform experiments and observe system design.
— The ocean fine-scale under sea ice-covered regions, their impact on air-ice-ocean fluxes and sea ice distribution and rheology remain enormous observational gaps for the Southern Ocean, particularly at the scale of sea ice leads, flows and polynyas. New technological innovation is needed to sample this harsh environment below, within and above sea ice at high-frequency and over weeks to months at a time. This requirement represents the very edge of our observational capability. Recent deployments of Seasonal Ice Mass Balance Buoys [109] are opening up opportunities to collect these observations within sea ice but more is needed to obtain systematic ocean vertical profiles beyond what is available from seal-tags and ships.
— Direct estimates of air-sea fluxes, such as by EC methods, is needed over regions of different conditions (high winds, large sea state), over regions expected to be ‘hot spots' for ocean interior ventilation (high ocean EKE, standing meanders) and in the sea ice-covered Southern Ocean. EC approaches are being tested on autonomous surface vehicles and surface drifting platforms that hold significant potential to expand these observations spatially and temporally.
— Critical to understanding the ocean mixed-layer and thus the fluxes of heat and CO_2_ between the ocean and the atmosphere is in constraining upper ocean turbulence. Most of the literature on turbulence in the surface boundary layer has been based on ship or laboratory-based observations ranging in duration from 2 to 10 days in shallow boundary layers and under modest wind forcing (e.g. [110]). These observations, which form the basis for mixing parameterizations, are made in very different conditions to the Southern Ocean where large mixed-layer biases remain a weakness for many models. The automation of turbulence profiling (e.g. direct measurements on gliders) offers opportunities to re-evaluate current scalings/parameterizations under a wide range of conditions (e.g. fine-scale gradients, waves and strong wind forcing).
— New satellites such as the Surface Water and Ocean Topography mission (SWOT, launched in December 2022; [111,112]) and planned missions, such as the Ocean Dynamical Surface Exchange with the Atmosphere (ODYSEA)/Waves and Current Mission (WaCM, [113]) and Sea surface Kinematics Multiscale monitoring (SKIM, [114]) can be related to *in situ* observations to provide new insights into fine-scale variability, energy and its distribution spatially and temporally. The ever-improving capability and need for satellites to measure variables required to estimate air-sea fluxes over the global ocean continues (e.g. J-OFURO3: [115]; OAFlux-HR: [116]). This will better our understanding of ocean-atmosphere interactions occurring at fine-scale and their influence on weather and climate.
— Finally, and possibly most critically, we require improved integration between high-resolution coupled model simulations and field observations to upscale the detailed view of *in situ* measurements and to understand the impact of fine-scale processes on large-scale climate and ecosystems. New data science techniques are becoming prominent, which will assist in this challenge. There is a need to develop high-resolution data assimilating models or state estimates, which are only feasible with higher densities of *in situ* measurements in the Southern Ocean.

There is an ever-growing capability of observing the ocean and lower atmosphere at the fine-scale. Improved sensor energy requirements and their miniaturization means that new variables (e.g. acoustics, nutrients, gases, biogeochemistry and microstructure) can be integrated onto autonomous vehicles that are becoming more robust, intelligent and improving their endurance via energy harvesting and enhanced battery capacity. Similar sensor additions are emerging for animal-borne approaches too (turtles, seals, fish) but we require cost and energy improvements in animal tag satellite data transmission to improve the current restriction of transmitting approximately 20 points per profile. New innovations that we do yet fully realize should be encouraged to close the observational gaps that we face today. Emerging adaptive sampling techniques (e.g. targeted regions, features or seasons), together with the ability for new satellite missions and models to resolve the fine-scale means we are at the cusp of providing a more holistic view of the various fine-scale processes that impact the transport of properties throughout the Southern Ocean and beyond, and the ability of the Southern Ocean to buffer the effects of climate change.

## Data Availability

This article has no additional data.
